# A300 ADVANCED HEPATIC ECHINOCOCCOSIS: A CASE REPORT

**DOI:** 10.1093/jcag/gwad061.300

**Published:** 2024-02-14

**Authors:** S Y Quan, M Wells

**Affiliations:** University of Alberta, Edmonton, AB, Canada; University of Alberta, Edmonton, AB, Canada

## Abstract

**Background:**

Alveolar echinococcosis (AE) is a rare and potentially fatal disease caused by the parasite echinococcus multilocularis (EM). Humans are intermediate hosts who become infected through contact with definitive hosts (foxes, wolves, coyotes, dogs and cats) and ingestion of contaminated food and water. AE primarily affects the liver but has the potential for local or metastatic spread. It has a clinical latency time of 5-15 years and resembles a malignancy in appearance and growth, posing a challenge in diagnosis and management. Treatment includes benzimidazole therapy and surgical resection, however, removal of the entire parasitic mass is not always possible.

**Aims:**

Present a case of hepatic AE involving the gallbladder, biliary tree, and spleen.

**Methods:**

Case report.

**Results:**

A 49-year-old farmer was admitted to a Saskatchewan hospital for a 2-week history of abdominal pain, jaundice, and progressive weight loss. Computed tomography (CT) of the abdomen and pelvis showed a necrotic mass in the gallbladder compromising the liver, splenic lesions and involvement of the biliary tree. The patient was given a preliminary diagnosis of metastatic cholangiocarcinoma and underwent a gallbladder biopsy which returned as echinococcus, confirmed with two liver biopsies and a positive EM nucleic acid test. He was then diagnosed with advanced hepatic AE with metastasis to the gallbladder, biliary tree, and spleen. The patient required a long-term hepatic drain and developed a hepatocutaneous fistula. While surgery is curative, his disease was too extensive for resection. He was started on Albendazole and received into our care for a liver transplant assessment 10 months later. Currently, there is limited literature on liver transplantation in advanced AE as it may not be curative. Additionally, subsequent immunosuppression may expedite residual parasite growth leading to aggressive disease recurrence. In this case, liver transplant would also require a splenectomy and excision of the hepatocutaneous fistula for complete parasite removal. Repeat CT of the abdomen and pelvis at thirteen months after the patient's initial hospitalization did not show disease progression. Given his stability on Albendazole, the added surgical complexity, and possibility of aggressive disease recurrence post liver-transplant, the multidisciplinary committee decided against liver transplant at this time. He continues to do well on Albendazole 15 months after initial presentation.

**Conclusions:**

The reported mortality rate of AE is ampersand:003E 90% within 10-15 years of diagnosis if untreated or inadequately treated. This case highlights the complexities in management of advanced hepatic AE and the importance of early diagnosis and treatment with radical surgery. Although not curative, benzimidazole therapy is effective in slowing disease progression in unresectable cases. Further studies are needed to assess the role of liver transplantation in advanced hepatic AE.

**Funding Agencies:**

None

